# Development of the workplace inclusion questionnaire (WIQ)

**DOI:** 10.1177/1403494821990241

**Published:** 2021-02-12

**Authors:** Vigdis Sveinsdottir, Tone Langjordet Johnsen, Tonje Fyhn, Jon Opsahl, Torill Helene Tveito, Aage Indahl, Hege Randi Eriksen, Silje Endresen Reme

**Affiliations:** 1 NORCE Norwegian Research Centre, Bergen, Norway; 2 Division of Physical Medicine and Rehabilitation, Vestfold Hospital Trust, Tønsberg, Norway; 3 Department of Health, Social and Welfare Studies, University of South-Eastern Norway, Horten, Norway; 4 Department of Sport and Physical Activity, Western Norway University of Applied Sciences, Bergen, Norway; 5 Department of Psychology, University of Oslo, Oslo, Norway

**Keywords:** Attitudes, prejudice, discrimination, stigma, vocational rehabilitation, workplace, workplace inclusion

## Abstract

**Aims:**

To develop a questionnaire to examine attitudes among employees and managers to include people with various health problems into their work group, and to test the questionnaire in one relevant population within the labour market.

**Methods:**

A questionnaire was developed through a process involving discussions in a scientific forum and pilot testing with group discussions. The final questionnaire, which was tested in a survey study of managers and employees in 33 Norwegian kindergartens (*N*=485), contained 10 short case stories followed by questions concerning workplace inclusion. The case stories described individuals with musculoskeletal and mental disorders, as well as individuals with potentially stigmatising behavioural history and lifestyle, and control cases. Risk ratios with 95% confidence intervals (CIs) were used to compare the case stories. Cases with high risk ratios had an increased risk of not being included compared to a control case.

**Results:**

Attitudes for workplace inclusion varied between the different case stories. Cases portraying mental illness had the highest risk ratios, indicating that employees and managers are less likely to include people with mental illness than people with musculoskeletal illness. Furthermore, unspecific or chronic illness had higher risk ratios than specific and acute illness. The most important barriers also varied between case stories.

**Conclusions:**

The workplace inclusion questionnaire fulfills the need for a quantitative measure of attitudes to include individuals with various health problems into the workplace. Comparison of risk ratios showed clear differences between case stories, indicating that the workplace inclusion questionnaire is a valuable tool to measure the variance in workplace inclusion.

## Background

A large part of the potential workforce is excluded from working life for different reasons. In Norway, 674,000 labour years were lost due to health problems or unemployment in 2018, corresponding to 18.9% of the working-age population [1]. The main reasons for sick leave and disability are related to unspecific musculoskeletal complaints such as low back pain and common mental disorders such as anxiety and depression [2]. Sick leave in Norway is highest in the health and social care sector, especially among employees in nursing homes and kindergartens [3]. The inclusive working life agreement (IA-agreement) [3] has made the workplace a priority setting for reducing sick leave and exclusion from working life among employees who are on sick leave or have impaired work capacity in Norway. The IA-agreement specifically focuses on industries and sectors with a large need and potential for sick leave reduction and preventive work environment efforts.

Work participation may be affected by social stigmatisation and the willingness of employers and employees to include individuals with, for example, various health problems in the workplace. As classically described by Goffman [4], social stigma involves discrediting individuals based on characteristics that are deemed socially undesirable. Core types of stigma may be grouped as stigma related to an individual’s demographic background, character and behaviour, or physical impairments [4]. With regard to stigma towards various health problems – which are the main focus in our study – these may fall within both of the two latter categories. Social stigma may be expressed as ignorance, prejudice and discrimination [5] and act as a barrier to employment that prevents individuals from staying in or even entering the labour market [6]. General surveys of employers’ attitudes and practices towards workers with disabilities often reflect favourable attitudes that may be biased by social desirability and employer self-selection [7], and despite expressing positive global attitudes, employers tend to be more negative when specific attitudes towards these workers are assessed [8]. Principal barriers to employing workers with disabilities may be a lack of awareness about disability and accommodation issues, concerns over potential expenses and fear of legal liabilities such as lawsuits or discrimination accusations [7].

Previous studies have shown that willingness to grant accommodation is greater when disability is caused by external factors rather than when it is attributed to the individual’s own behaviour [9], and that workers with physical disabilities are viewed more positively than workers with intellectual or psychiatric disabilities [8, 10]. Even so, stigma towards physical disabilities such as back pain is still a barrier for workplace inclusion [11], but stigma towards mental illness is especially widespread [5]. For people with severe mental illness the rates of both anticipated and experienced discrimination are high across countries and labour markets [12]. In addition to concerns about clinical and work performance factors, employers report negative beliefs about personal factors regarding the employment of people with mental illness, including lack of trust and safety issues when working with vulnerable groups such as children and the elderly [13]. There is, however, little knowledge about how stigma or attitudes towards inclusion at the workplace varies between different diagnoses. Furthermore, distinctions between specific and acute as opposed to unspecific and chronic health conditions may be of importance in this context. Previous qualitative research has indicated that workers find it more difficult to accept and accommodate colleagues who have longstanding and unspecific conditions, as opposed to more specific and short-term health problems [14]. In addition to stigma towards health problems, another core type of stigma is that associated with behavioural history which may or may not represent health problems, such as substance use and lifestyle choices that are perceived as flaws to the individual’s character [4].

Familiarity and experience in working with people with various challenges is associated with more favourable attitudes [15]. This may be explained by the contact hypothesis, as described by Allport [16], in which interaction with members of another group may decrease prejudice and lead to more favourable attitudes that are generalised beyond the immediate situation [17]. Moreover, interventions aiming to improve communication about health concerns at the workplace, debunk common myths, provide reassurance and reduce fear related to the workers’ symptoms, have been shown to be effective in reducing sick leave (e.g. Odeen et al.) [18], possibly due to changes in attitudes and higher acceptance of people returning to work in spite of their health problems.

Although there exists a fair amount of studies that examine the employability of, or attitudes towards, different groups – they tend to examine only a few health problems, or not to distinguish between different types of health problems at all [19, 20]. Previous measures do thereby not provide the opportunity to compare how different health problems are rated at the workplace. Furthermore, questionnaires measuring attitudes towards people with mental illness commonly have a broad perspective on social inclusion, only sometimes including work [21–23]. There is therefore a need for an operationalisation of the concept of attitudes towards workplace inclusion, and a measure to investigate these attitudes towards individuals across a broader spectre of health problems.

In this paper, we operationalise workplace inclusion as a concept to describe attitudes about how different individuals are considered to fit into a work group, whether the individual is being hired or is already employed. If members of a group consider an individual not to fit in, it is likely to affect their inclusion practices. Inclusion is a complex concept that may be experienced differently across situations and may operate at the individual, interpersonal, group, organisational and societal level. By narrowing this down to an employer’s or employee’s own perceptions about how well different individuals fit into their own job environment, we here seek to give insight into the workplace inclusion of various cases representing people with different health problems. These are again compared and contrasted to cases with a potentially stigmatising behavioural history, and to control cases without such a history or problems.

## Aims

The first aim of the study was to develop a questionnaire to assess attitudes towards the workplace inclusion of individuals with different health problems, with the opportunity to compare attitudes across different cases and various sectors in the labour market. The choice of different health problems was carefully selected from the most prevalent diagnostic groups in the workers’ compensation system, and aimed to include both specific and acute, as well as unspecific and chronic health problems. For comparability, the workplace inclusion questionnaire (WIQ) also includes descriptions of individuals with a different behavioural history and lifestyle, and control cases with no such history or health problems.

The second aim was to test the questionnaire in one common and relevant sector of working life. In this study, we chose the kindergarten sector, as it represents a large and important part of the Norwegian labour market with a high level of health problems among employees [3].

## Methods

### Part 1: Development of the WIQ

#### Development process

The WIQ was initially drafted as a scale consisting of simple items presenting cases with different behavioural or demographic characteristics, health complaints or diagnoses varying in prevalence and severity, to be rated on a Likert scale based on how well each case was considered to fit into the respondent’s work group. The goal was to describe a broad spectrum of cases including the most common reasons for sick leave and disability. The questionnaire was modified through a process of critical discussion with researchers within health and social sciences. Describing cases in short sentences or just using diagnostic names was deemed too simplistic. To create valid case stories, information about job qualification, age and gender was combined with a description of diagnostic criteria based on the International Classification of Diseases version 10 (ICD-10) or behavioural and demographic characteristics [24]. The idea was to provide respondents with an understanding of each condition, without mention of the diagnosis.

We developed 10 short case stories, describing people with various musculoskeletal, mental and behavioural problems which represent the major reasons for sick leave and disability in Norway [2], as well as different social groups, without health problems, but with specific behavioural or demographic characteristics. The gender of the cases was randomly selected, and included five men and five women. The cases were given common names corresponding to name trends at their time of birth, and each consisted of a few sentences describing the specific characteristics or health or behavioural problems and functioning of each case. In order to avoid qualifications and age interfering with the evaluations of the cases, all cases were described as having the formal qualifications necessary for the job and to be in their 30–40s, with the exception of one case presented as an older worker.

#### Pilot testing with group discussion

We performed a pilot test in order to ensure that the questionnaire was meaningful and understandable, and addressed authentic problems and situations for each case. The questionnaire was distributed among two groups of managers and human resource employees from different sectors of the labour market. All participants (*N*=40, mean age 47.4 years (standard deviation 9.4), 90% women, 42.5% with hiring responsibilities) were asked to indicate how well they considered each case to fit into their work group, and if relevant, to state the main reason why the case did not fit into the group. After the pilot testing, the participants were invited to discuss the questionnaire with the researchers. Discussions involved social desirability bias, the difference between making accommodations for existing colleagues versus hiring of new employees, and the risk of increased workload for other employees. Based on the feedback provided in the pilot study, subsequent adaptions were made.

#### Case stories

Six cases described health conditions corresponding to the diagnostic criteria in the ICD-10 for musculoskeletal, mental and behavioural disorders.

- Chronic back pain (M54.5) (Lisa)
- Spine fracture (T08) (Matthew)
- Mild to moderate depressive episode (F32.0-1) with symptoms of anxiety (Jennifer)
- Schizophrenia with stable deficit (F20) (Michael)
- Hyperkinetic disorder (F90) (Ashley), corresponding to attention deficit hyperactivity disorder in ICD version 11
- Somatisation disorder (F45.0) (Melissa).

The four remaining cases described common social groups without current health problems.

- Previous drug addiction (Christopher)
- Unhealthy lifestyle (John)
- Single mother with young child (Sarah)
- Older worker with possibility for early retirement (James).

The cases with previous drug addiction and unhealthy lifestyle were considered as cases who might be stigmatised due to behavioural history and lifestyle. The single mother and older worker were considered as being control cases.

#### Questions

Following each case story, the respondents were asked two questions: (a) ‘In an ideal world, how do you think N.N. would fit into your work group?’; (b) ‘Given the current circumstances, how do you think N.N. fits into your work group?’ (i.e. workplace inclusion). The answers were scored on a 5-point Likert scale: 1 = very poorly; 2 = quite poorly; 3 = neither poorly nor well; 4 = quite well; and 5 = very well. The two-question solution with ideal and current circumstances was made with the intention of giving respondents an opportunity to express socially desirable responses, before asking about actual attitudes to include each case. The third question was intended to measure barriers: ‘If N.N. does not fit quite/very well into your work group: What is the main reason?’ The possible responses were: ‘need for accommodation’, ‘economic consequences’, ‘collaboration/interaction with colleagues’, ‘ability to provide service’, ‘increased workload for colleagues’, ‘work capacity’, ‘work ability’, and an open response category. Finally, the respondents were asked to answer yes or no to the question: ‘Do you have any experience with colleagues or employees like N.N.?’

### Part 2: Testing of the WIQ

#### Study population and data collection

The questionnaire was distributed to managers and employees in 33 Norwegian municipal kindergartens, using electronic survey software (Qualtrics). The participants received written information and gave consent by answering and submitting the survey, and 485 employees finished the survey. Each respondent received and rated a random selection of five out of the 10 cases. The questionnaire was anonymous and only the research team had access to the responses. The study was approved by the Norwegian Data Protection Official for Research (registration no. 34934/3/KS).

#### Statistical analysis

Descriptive data analyses were performed for background characteristics of the participants and workplace inclusion variables. Boxplots with means and 95% CIs were computed for visual comparison of the various case stories on workplace inclusion. The variables measuring attitudes about how well the different case stories fit into the work group in ideal and current circumstances were dichotomised into 0 (very poorly, quite poorly, and neither poorly nor well) or 1 (quite well and very well) before analyses were performed. Risk ratios with 95% confidence intervals were then calculated to investigate differences in the relative risk for workplace inclusion between the various cases using the older worker as a control/reference case, and also between ideal and current circumstances for each case story. Differences in workplace inclusion between respondents with versus without previous experience with similar cases, and between respondents with versus without hiring responsibilities, were tested using chi square tests. Descriptive data analyses were performed for the barriers reported for each case, and open-ended responses were categorised using thematic analysis, as described by Joffe and Yardley [25]. The categorisation was performed independently by two of the authors, and any inconsistencies were discussed until consensus was reached.

## Results

The majority of the participants were older than 40 years (*n*=276, 59%) and the most common education level was 1–4 years of college or university (*n*=201, 43%). Forty-five participants (9.7%) had responsibilities for the selection and hiring of new staff. Due to the low number of men in Norwegian kindergartens (approximately 11%) [26] we did not ask about gender in this sample.

### Comparison of case stories

**Figure 1.**
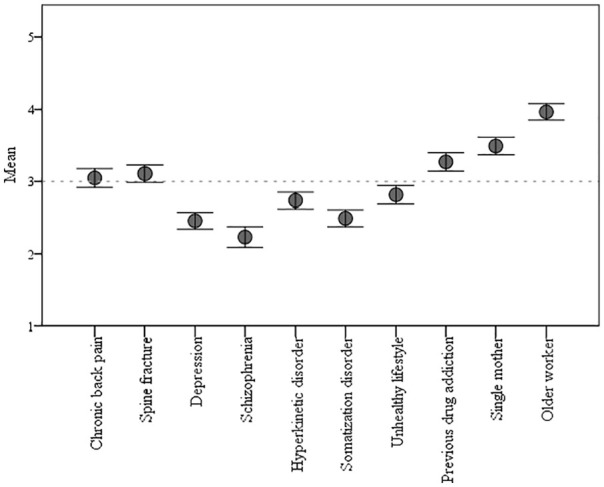
Mean and 95% confidence interval for workplace inclusion of each case story on a scale from 1 (very poorly) to 5 (very well): ‘Given the current circumstances, how do you think person N.N. fits into your work group?’.

The older worker had the highest and most favourable mean score on workplace inclusion, followed by the single mother and the case with previous drug addiction (see Figure 1). The cases with spine fracture, chronic back pain, unhealthy lifestyle and hyperkinetic disorder were concentrated around the centre of the scale. The three cases with the lowest and least favourable mean scores were somatisation disorder, depression and schizophrenia.

The distribution of positive, neutral and negative responses for each case story showed that more than half of the respondents were positive regarding the older worker and single mother who were considered as being control cases, and more than half of the respondents were negative towards schizophrenia, depression and somatisation disorder (see Table I).

**Table I.** Number and percentage of responses for each case story in both ideal and current circumstances, and how many who had previous experience with the case in question.

	Total *n*	Very poorly				Quite poorly				Neither				Quite well				Very well				Experience	
		Ideal		Current		Ideal		Current		Ideal		Current		Ideal		Current		Ideal		Current			
		*n*	%	*n*	%	*n*	%	*n*	%	*n*	%	*n*	%	*n*	%	*n*	%	*n*	%	*n*	%	*n*	%
Chronic back pain	230/227	15	6.5	12	5.3	56	24.3	58	25.6	67	29.1	76	33.5	80	34.8	69	30.4	12	5.2	12	5.3	168	73.7
Spine fracture	233/231	10	4.3	10	4.3	39	16.7	49	21.2	94	40.3	88	38.1	81	34.8	74	32	9	3.9	10	4.3	76	34.2
Depression	231/231	33	14.3	35	15.2	84	36.4	84	36.4	78	33.8	84	36.4	33	14.3	27	11.7	3	1.3	1	0.4	106	46.9
Schizophrenia	220/214	63	28.6	64	29.9	72	32.7	70	32.7	49	22.3	50	23.4	31	14.1	26	12.1	5	2.3	4	1.9	23	10.7
Hyperkinetic disorder	228/224	15	6.6	16	7.1	71	31.1	74	33	93	40.8	88	39.3	46	20.2	45	20.1	3	1.3	1	0.4	126	55.8
Somatisation disorder	231/229	27	11.7	29	12.7	80	34.6	89	38.9	91	39.4	84	36.7	28	12.1	23	10.1	5	2.2	4	1.7	132	59.2
Unhealthy lifestyle	230/226	23	10	23	10.2	60	26.1	57	25.2	87	37.8	93	41.2	50	21.7	45	19.9	10	4.3	8	3.6	96	42.9
Previous drug addiction	226/225	13	5.8	12	5.3	31	13.7	30	13.3	79	35	87	38.7	80	35.4	77	34.2	23	10.2	19	8.4	41	18.3
Single mother	232/230	5	2.2	6	2.6	19	8.2	28	12.2	67	28.9	71	30.9	103	44.4	97	42.2	38	16.4	28	12.2	183	81.3
Older worker	233/233	4	1.7	4	1.7	9	3.9	10	4.3	13	15	41	17.6	113	48.5	113	48.5	72	30.9	65	27.9	118	51.3

The probability of being rated as a person who fits well into the respondents’ work group differed between the various case stories. When compared with the older worker, all the other case stories had a lower probability of receiving a favourable rating (see Table II). The person with a somatisation disorder and the person with depression were more than six times as likely to be rated less favourably, the person with schizophrenia was over five times as likely to be rated less favourably, and the person with hyperkinetic disorder was almost four times as likely to be rated less favourably than the older worker. The person with an unhealthy lifestyle had over three times the probability of a less favourable rating than the older worker, and both the person having a spine fracture and the person with chronic back pain had twice the probability of a less favourable rating. Finally, the person having a previous drug addiction and the single mother had a 79% and 40% increased probability of receiving a less favourable rating than the older worker, respectively.

**Table II.** Percentage willing to include each case story at their workplace and the risk ratio for not being included when compared to the older worker.

	Willing to include %	RR	95% CI low	95% CI high
Somatisation disorder	11.7	6.48	4.51	9.30
Depression	12.1	6.30	4.42	8.98
Schizophrenia	14.0	5.45	3.88	7.65
Hyperkinetic disorder	20.5	3.72	2.85	4.86
Unhealthy lifestyle	23.4	3.26	2.55	4.17
Chronic back pain	35.7	2.14	1.77	2.59
Spine fracture	36.3	2.10	1.75	2.53
Previous drug addiction	42.6	1.79	1.51	2.12
Single mother	54.4	1.41	1.22	1.61
Older worker (ref)	76.4	1		

CI: confidence interval; RR: risk ratio.

### Previous experience

The respondents who reported previous experience with a colleague or employee resembling the cases of spine fracture (χ^2^ (1, *n*=220) = 0.015, *P*=0.012, phi = −0.010), unhealthy life style (χ^2^ (1, *n*=217) = 0.005, *P*=0.005, phi = −0.201), single mother (χ^2^ (1, *n*=222) = 0.031, *P*=0.024, phi = −0.157), or older worker (χ^2^ (1, *n*=228) = 0.001, *P*=0.001, phi = −0.237) were more positive towards including the respective cases at their workplace, but the effect sizes were small. Results for the remaining cases were not statistically significant.

### Ideal and current circumstances

Differences in the probability for workplace inclusion when considering ideal or current circumstances were small and not statistically significant (see Table III).

**Table III.** Percentage willing to include and the risk ratio for being included in ideal circumstances compared to current circumstances.

	Ideal	Current	RR	95% CI low	95% CI high
	% willing to include	% willing to include			
Chronic back pain	40.0	35.7	1.12	0.89	1.42
Spine fracture	38.7	36.3	1.06	0.84	1.34
Depression	15.6	12.1	1.29	0.81	2.03
Schizophrenia	16.4	14.0	1.17	0.75	1.82
Hyperkinetic disorder	21.5	20.5	1.05	0.73	1.50
Somatisation disorder	14.3	11.7	1.21	0.75	1.95
Unhealthy lifestyle	26.0	23.4	1.11	0.81	1.53
Previous drug addiction	45.6	42.6	1.07	0.87	1.32
Single mother	60.8	54.4	1.12	0.96	1.31
Older worker	79.4	76.4	1.04	0.94	1.14

CI: confidence interval; RR: risk ratio.

### Hiring responsibilities

There were no significant differences in ratings between those with or without hiring responsibilities, with the exception of one case. The respondents with hiring responsibilities were significantly more positive towards including the case with the single mother, but the effect size was small (χ^2^ (1, *n*=224) = 0.042, *P*=0.025, phi = −0.151).

### Barriers

Increased workload for colleagues was the most commonly reported barrier for chronic back pain, spine fracture, somatisation disorder and the single mother. Work capacity was the most commonly reported barrier for the cases describing an unhealthy lifestyle, the older worker and also spine fracture. Collaboration/interaction with colleagues was the main barrier for the case with hyperkinetic disorder and work ability was the main barrier for the cases describing previous drug addiction, depression and schizophrenia (see Table IV).

**Table IV.** Barriers for workplace inclusion reported for each case story.

	Total *n*	Need for accommodation	Economic consequences	Collaboration with colleagues	Ability to provide service	Increased workload for colleagues	Work capacity	Work ability	Other
Chronic back pain	94	16.0%	7.4%	–	1.1%	**28.7%**	14.9%	19.1%	12.8%
Spine fracture	145	18.6%	5.5%	0.7%	2.1%	**27.6%**	**27.6%**	14.5%	3.4%
Depression	123	5.7%	4.1%	21.1%	11.4%	13.0%	12.2%	**22.8%**	9.8%
Schizophrenia	179	8.4%	2.2%	15.6%	9.5%	7.3%	8.4%	**25.7%**	22.9%
Hyperkinetic disorder	169	2.4%	0.6%	**50.3%**	5.3%	18.9%	4.1%	10.7%	7.7%
Somatisation disorder	192	5.7%	9.4%	5.2%	3.1%	**35.9%**	21.9%	15.6%	3.1%
Unhealthy lifestyle	158	5.1%	10.1%	0.6%	8.2%	17.7%	**32.9%**	15.8%	9.5%
Previous drug addiction	112	8.9%	4.5%	3.6%	7.1%	9.8%	12.5%	**29.5%**	24.1%
Single mother	101	2.0%	16.8%	2.0%	–	**48.5%**	13.9%	6.9%	9.9%
Older worker	55	1.8%	5.5%	12.7%	9.1%	9.1%	**29.1%**	12.7%	20.0%

Numbers in bold indicate the most frequently reported barrier per case story.

#### Open-ended responses to barriers

For chronic back pain, respondents had concerns about practical issues related to job-specific tasks, unpredictability and worries about sick leave, while for spine fracture mainly practical issues related to job-specific tasks were reported. For the case with depressive symptoms there were worries about caring responsibilities, working environment and lack of energy/positivity. The case with schizophrenia yielded the largest number of open responses, and concerns were related to safety risks and possible danger and unpredictability (e.g. ‘Unsure if he is stable and whether he is going to be a crazy axe-murderer’) and practical issues or job-specific tasks in dealing with children. For hyperkinetic disorder, concerns were related to issues of unpredictability, unrest and interaction with children. For somatisation disorder, respondents had concerns about excessive complaining about health problems and worries about sick leave. For the case with an unhealthy lifestyle, respondents had concerns about willingness to change, level of physical activity, working environment and interaction with children, while for the case with previous drug addiction, concerns were related to mistrust, risk of relapse and the working environment, but some responses expressed possible advantages. The single mother raised concerns about sick leave and staffing, while the older worker raised concerns about interaction with children and colleagues, and also age.

## Discussion

### Main results

The first aim of the study was to develop a new questionnaire to measure workplace inclusion of various groups that may face stigma due to their health problems, as compared to individuals with a different behavioural history and lifestyle, and control cases. Ten case stories were developed. The first six cases described people with musculoskeletal and mental disorders, which represent the main diagnostic groups on sick leave and long-term disability in Norway [2]. The cases included both specific and acute health problems and unspecific and chronic health problems. These were chronic back pain, spine fracture, depression, schizophrenia, hyperkinetic disorder and somatisation disorder. The remaining four cases described individuals without current health problems but with a potentially stigmatising lifestyle and behavioural history (unhealthy lifestyle and previous drug addiction), and control cases without such health problems or histories (a single mother and an older worker). The questionnaire primarily measured attitudes to, and barriers for, including each case at the workplace.

The second aim of the study was to test the questionnaire in one common and relevant sector of the Norwegian labour market. We chose the kindergarten sector as it is a large and important part of the Norwegian labour market, with high sick leave and a large potential for preventive work environment factors [3]. The results from the study of managers and employees in Norwegian kindergartens showed that attitudes to include people into one’s working environment (workplace inclusion) varied for the different case stories. The three cases that were rated most favourably all represent different social groups in which illness or current health problems are not reported, including the control cases. Both cases representing musculoskeletal illness/injury were rated relatively high, while the lowest rated cases involved mental illnesses. These results are in concordance with previous literature on stigma towards workers with mental illness [27], and especially with regard to severe mental illness [12]. The findings indicate a need for efforts targeting stigma towards employees with mental illness in the Norwegian kindergarten setting, and underline the importance of interventions aiming to improve communication and increase acceptance of co-workers facing these health problems. Furthermore, specific barriers for inclusion in this context were specified. The barriers reported by respondents who rated the cases describing mental illnesses negatively showed that increased workload for colleagues, work ability and collaboration/interaction with colleagues were the major concerns. A large number of open responses were also provided. The responses for the case of schizophrenia were especially numerous and expressed worry about safety risks and danger. It should, however, be kept in mind that the working population investigated in this specific study concerns the care for children, a particularly vulnerable context that may further exacerbate worry regarding safety issues [13], and many responses were specifically related to perceived incompatibility with this line of work.

Case stories with unspecific or chronic illness, such as depression, hyperkinetic disorder and somatisation disorder, were rated less favourably than the acute and specific case of spine fracture. These findings are in line with previous qualitative research [14], and may be explained by stigma towards symptoms that are long term, difficult to define and have unclear aetiology, as opposed to illness that is of a specific and acute nature. Furthermore, somatisation disorder involves multiple, frequently changing and unexplained symptoms, which may have further exacerbated such stigma. Chronic back pain was, however, rated more favourably than the other cases of unspecific and chronic illness, indicating that musculoskeletal illness in general may have been less susceptible to this stigma.

### Previous experiences

For four of the cases, previous experience with similar colleagues or employees was of relevance for workplace inclusion, in line with the contact hypothesis and previous research showing that intergroup contact may reduce prejudice and thus promote acceptance [16, 17]. Those who had previous experience with colleagues with spine fracture, unhealthy lifestyles, single mothers, or older workers, rated these cases more favourably. The three latter cases represent groups without current health problems and include both of the control cases, and the acute and specific case of spine fracture was rated highest among the cases describing various health challenges. This suggests that previous experience had a larger influence on the inclusion of groups that are less prone to stigmatisation.

The existing research on familiarity with mental illness indicates that if there is a relationship between previous experience and attitudes, it is likely to be positive [15]. In the current study, it is possible that low statistical power in the case story of schizophrenia may have been responsible for the lack of significance, because very few workers had previous experience with colleagues with this particular disorder.

We only measured whether respondents had any experience with colleagues and/or employees similar to the various case stories, thus including both positive and negative, extensive and brief experiences. Quality of contact may, however, be a more important predictor for attitudes than knowledge and quantity of contact [28] even though these are interrelated constructs. Quality and type of contact was not assessed, and the lack of such nuances could explain the lack of significant findings among the remaining cases. The use of more refined variables for measuring contact may be necessary in order to detect such variances [29], although this was not the primary focus of the current study and may be too comprehensive in the context of the WIQ.

### Attitudes, social desirability and behaviour

Behaviour is a notoriously difficult construct to assess, and as pointed out by Corrigan et al. [30] most studies do not have the resources necessary to observe actual responses after measuring attitudes, and in many cases such observations would not be practically feasible. Self-report measures such as these represent behavioural intentions which may be inconsistent with actual actions. Steps taken to reduce social desirability in the current study involved the addition of an item asking about ideal circumstances, providing participants with a chance to express socially desirable responses before considering how well each case would fit in given the current situation at their workplace. Ratings of the cases when considering ideal circumstances were consistently higher than scores in the current circumstances, but the differences were very small and not statistically significant. The benefit of including this item may thus be limited.

### Methodological considerations

Due to restrictions in the format of the study, each respondent only received a random selection of five out of the 10 case stories, thereby reducing statistical strength. Still, we argue that the data material is sufficient to respond to the study aims. As the study sample consisted of employees in Norwegian kindergartens with a majority of women, participants were not asked to specify gender or exact age due to the risk of individual participants being identified, which hinders analyses of subgroups. We recommend that future studies consider these limitations in accordance with their objectives.

#### Reliability and validity

The nature of the WIQ precludes common tests of reliability and validity due to the unique quality of each case story and the fact that cases do not form subscales or produce a sum score. The design of the current study did not allow test–retesting. Content validity was evaluated through pilot testing and group discussions about the relevance and meaningfulness of the case stories.

#### Generalisability

The WIQ was designed as a global measure of workplace inclusion, which in principle can be used across all sectors of the labour market as it is not occupation specific. The current study investigated attitudes for workplace inclusion among managers and employees in kindergartens, and many of the reported barriers, especially those concerning mental health problems, were specifically related to concerns for the children. While the nature of the study sample causes clear limitations to the generalisability of the results, generalising across different parts of the labour market is not the aim of the WIQ. It is to be expected that workplace inclusion of different individuals may differ for various types of occupations and working environments and the aim of the study was rather to develop an instrument to investigate this phenomenon in different contexts.

#### Possible gender effects

While the case stories represent individuals in their 30–40s and are presented with equal formal qualifications, responses may be influenced by whether individual case stories are represented as male or female. Investigations of gender bias, for example, by interchanging names of male and female case stories, are therefore warranted.

#### Further improvements and updates to the WIQ

Adjustments made to the questionnaire after the survey study include simplifications and improvements to questions and categories of barriers, and are included in the version provided in Supplemental material 1. The barrier ‘work ability’ was removed as it is incorporated into several of the other categories, and because all cases were described as having the needed qualifications for the job. ‘Ability to provide service’ was changed to ‘collaboration/interaction with others’ in order to increase generalisation across different types of workplaces. The question regarding ideal circumstances was removed from the questionnaire due to the non-significant differences between this item and the item asking about current circumstances, and thereby low perceived benefits, as well as to shorten the questionnaire.

Adding supplementary case stories representing additional groups of interest that may face stigmatisation is relevant in future developments of the questionnaire. Pertaining to Goffman’s descriptions of core types of stigma [4], these may include for example different cultural backgrounds, physical impairments, or criminal history. The use of selected case stories may also be sufficient to answer relevant hypotheses in certain contexts, thereby shortening the questionnaire.

The target group of the questionnaire could further be adapted to include not only employees and managers, but other important stakeholders in facilitating work participation, such as caseworkers in labour and welfare administration and vocational rehabilitation workers.

### Implications and relevance

Previous studies investigating employability and attitudes towards different groups have examined a few specific health problems or not distinguished between specific types of health problems at all, and do commonly not have a specific focus on the work context [19–23]. The WIQ adds to the existing knowledge in the field by providing a way to quantitively measure how people who may face stigmatisation due to a range of different health problems or characteristics related to behaviour and lifestyle are perceived to fit into a workplace, while keeping job qualification constant. The WIQ is a flexible measure in which the gender and age of case stories may be changed, depending on the aim of the study (e.g. to investigate gender effects or make all demographic factors equal for comparability reasons). The WIQ focuses on a broad range of health-related and social characteristics, and may be used to investigate differences in workplace inclusion across groups and diagnoses. The use of the WIQ across different working environments and sectors of the labour market will accumulate important knowledge about which individuals are more likely to be marginalised in different work settings. This information will be useful for employers as well as researchers and policy-makers in assessing where efforts should be placed to target stigma in working life, and furthermore to test the effect of interventions aiming to increase the workplace inclusion of people with various health problems. If interventions aimed at improving knowledge and attitudes about different stigmatised groups can influence these perceptions, it is likely to have a positive influence on inclusive practices. To reach the public aim of a more inclusive working life, we are in need of workplaces that do not have a restricted view of workplace inclusion. The questionnaire is currently being tested in different populations across a broad spectre of industries, and in a randomised controlled trial of a workplace intervention targeting employees’ beliefs about musculoskeletal and mental health complaints.

## Conclusions

The WIQ fulfills the need for a quantitative measure of inclusion in a workplace setting, across a broader spectre of health problems as well as other characteristics that may lead to stigma in working life. The questionnaire was tested in one relevant sector of working life, and discriminated between different case stories. Comparison of risk ratios showed that the cases describing persons with mental illness had the lowest probability for workplace inclusion in this context, and highlights a need for efforts targeting stigma and specific barriers for inclusion of employees with mental illness in the Norwegian kindergarten sector. Results are in accordance with previous literature on stigma towards mental illness, which may prevent vocational rehabilitation and lead to exclusion from the labour market.

## Supplemental Material

sj-pdf-1-sjp-10.1177_1403494821990241 – Supplemental material for Development of the workplace inclusion questionnaire (WIQ) Click here for additional data file. Supplemental material, sj-pdf-1-sjp-10.1177_1403494821990241 for Development of the workplace inclusion questionnaire (WIQ) by Vigdis Sveinsdottir, Tone Langjordet Johnsen, Tonje Fyhn, Jon Opsahl, Torill Helene Tveito, Aage Indahl, Hege Randi Eriksen and Silje Endresen Reme in Scandinavian Journal of Public Health
