# Supplementary material for: Development of the workplace inclusion questionnaire (WIQ)
Source: Scand J Public Health. 2021 Feb 12;50(3):371–80. doi: 10.1177/1403494821990241 (PMC9096575; doi:10.1177/1403494821990241)
Supplement: sj-pdf-1-sjp-10.1177_1403494821990241 – Supplemental material for Development of the workplace inclusion questionnaire (WIQ) [file sj-pdf-1-sjp-10.1177_1403494821990241.pdf]

Supplementary material 1

Title: The Workplace Inclusion Questionnaire (WIQ)

Authors: Sveinsdottir V*, Johnsen TL*, Fyhn T, Opsahl J, Tveito TH, Indahl A, Eriksen HR, Reme SE.

*Co-first authorship. These authors contributed equally to this work.

We will now describe a few different people. We want to know how well you think that each person fits into your work group. There are no right or wrong answers, we are interested in your personal opinions. Please answer as honestly as possible, and remember that your answers are anonymous.

Lisa

Lisa is a woman in her mid-forties, who has the qualifications required for the job. During the last few years, she has experienced pain and stiffness in her lower back for longer periods of time. Despite several medical examinations, it has not been possible to determine the cause or find any organic explanation for the complaints. The pain can be very intense and it gets worse after longer periods of sitting or standing still, but she can often find an activity or position that relieves the pain, in combination with non-prescription pain killers.

a) Given the current circumstances, how do you think Lisa fits into your work group?

Very poorly	Quite poorly	Neither poorly nor well	Quite well	Very well
☐1	☐2	☐3	☐4	☐5

b) If Lisa does not fit quite/very well into your work group: What is the main reason?
(Please select the one most important reason)

☐1 Need for accommodation
☐2 Economic consequences
☐3 Collaboration/interaction with colleagues
☐4 Collaboration/interaction with others
☐5 Increased workload for colleagues
☐6 Work capacity
☐7 Other: ____

c) Do you have any experience with colleagues/employees like Lisa?
☐1 Yes
☐2 No

James

James is a 62-year-old man. He has the qualifications needed for the job, and is in normal good health and shape compared to others in his age group. He has considered retiring, but concluded that he wants to work for a few more years.

a) Given the current circumstances, how do you think James fits into your work group?

Very poorly	Quite poorly	Neither poorly nor well	Quite well	Very well
☐1	☐2	☐3	☐4	☐5

b) If James does not fit quite/very well into your work group: What is the main reason?
(Please select the one most important reason)

☐1 Need for accommodation
☐2 Economic consequences
☐3 Collaboration/interaction with colleagues
☐4 Collaboration/interaction with others
☐5 Increased workload for colleagues
☐6 Work capacity
☐7 Other: ____

c) Do you have any experience with colleagues/employees like James?
☐[1] Yes
☐[2] No

Jennifer

Jennifer is a 35-year-old woman is in normal good health and has got the qualifications needed for the job. Recently, Jennifer has been feeling down, anxious and has had problems sleeping. She is not participating in any regular leisure activities or hobbies, and spends most of her time by herself. Jennifer often feels that she lacks energy, she does not put much effort into her own appearance, and tends to be pessimistic about the future.

a) Given the current circumstances, how do you think Jennifer fits into your work reason?

Very poorly	Quite poorly	Neither poorly nor well	Quite well	Very well
☐[1]	☐[2]	☐[3]	☐[4]	☐[5]

b) If Jennifer does not fit quite/very well into your work group: What is the main barrier? (Please select the one most important reason)
☐[1] Need for accommodation
☐[2] Economic consequences
☐[3] Collaboration/interaction with colleagues
☐[4] Collaboration/interaction with others
☐[5] Increased workload for colleagues
☐[6] Work capacity
☐[7] Other: ____

c) Do you have any experience with colleagues/employees like Jennifer?
☐[1] Yes
☐[2] No

Ashley

Ashley is a very active woman in the beginning of her thirties, who has the qualifications needed for the job. She tends to talk a lot, talks fast, and she seems positive and highly engaged. Despite this, Ashley has a tendency to jump from task to task without finishing what she started. She gets impatient easily, and at times it might seem like she does not pay attention. She can have trouble concentrating for longer periods of time. Growing up, Ashley had trouble behaving the way her parents and school expected her to, and had a tendency to break norms and rules, but she has better control of these things today.

a) Given the current circumstances, how do you think Ashley fits into your work group?

Very poorly	Quite poorly	Neither poorly nor well	Quite well	Very well
☐[1]	☐[2]	☐[3]	☐[4]	☐[5]

b) If Ashley does not fit quite/very well into your work group: What is the main reason? (Please select the one most important reason)

☐1 Need for accommodation
☐2 Economic consequences
☐3 Collaboration/interaction with colleagues
☐4 Collaboration/interaction with others
☐5 Increased workload for colleagues
☐6 Work capacity
☐7 Other: ____

c) Do you have any experience with colleagues/employees like Ashley?

☐1 Yes
☐2 No

Christopher

Christopher is a 30-year-old who has the qualifications needed for the job. He is a former drug addict, and he is looking for a fresh start in working life. During his youth, he drank a lot of alcohol, smoked cannabis and experimented with different narcotics such as amphetamine and cocaine. After being included in a drug rehabilitation program, he got back to school. He has been clean for two years and his criminal record shows that he has never been involved in any acts of violence or sexual abuse.

a) Given the current circumstances, how do you think Christopher fits into your work group?

Very poorly	Quite poorly	Neither poorly nor well	Quite well	Very well
☐1	☐2	☐3	☐4	☐5

b) If Christopher does not fit quite/very well into your work group: What is the main reason? (Please select the one most important reason)

☐1 Need for accommodation
☐2 Economic consequences
☐3 Collaboration/interaction with colleagues
☐4 Collaboration/interaction with others
☐5 Increased workload for colleagues
☐6 Work capacity
☐7 Other: ____

c) Do you have any experience with colleagues/employees like Christopher?

☐1 Yes
☐2 No

Michael

Michael is a 42-year-old man who has the qualifications needed for the job. Occasionally, he hears voices that comment hos everyday actions. Now and then he also gets the impression that people around him try to control his thoughts, which can lead him to say strange things or get annoyed. These symptoms present themselves in periods lasting a few weeks at a time, but are otherwise completely absent. Today, Michael is taking medications that give him good control of his symptoms. Michael is otherwise conscious and aware, and his intelligence is normal. There have not been any findings of organic brain disease, and he has no substance abuse problems. Michael's mother had the same condition, so there is reason to believe that the symptoms might be genetic.

a) Given the current circumstances, how do you think Michael fits into your work group?

Very poorly	Quite poorly	Neither poorly nor well	Quite well	Very well
☐1	☐2	☐3	☐4	☐5

b) If Michael does not fit quite/very well into your work group: What is the main reason? (Please select the one most important reason)

☐1 Need for accommodation
☐2 Economic consequences
☐3 Collaboration/interaction with colleagues
☐4 Collaboration/interaction with others
☐5 Increased workload for colleagues
☐6 Work capacity
☐7 Other: ____

c) Do you have any experience with colleagues/employees like Michael?

☐1 Yes
☐2 No

Matthew

Matthew is a man in his thirties, who has the qualifications needed and is in normal good health. He suffered a fractured spine in a traffic accident 6 months ago, and needed to have surgery using screws to stabilize his spine. He was in a lot of pain after the surgery, but was told that this was normal and got prescriptions for painkillers. He is currently getting treatment and training from a physical therapist. The therapist recommends Matthew to get back to normal activity, while avoiding team sports or competitions for a few more months.

a) Given the current circumstances, how do you think Matthew fits into your work group?

Very poorly	Quite poorly	Neither poorly nor well	Quite well	Very well
☐1	☐2	☐3	☐4	☐5

b) If Matthew does not fit quite/very well into your work group: What is the main reason? (Please select the one most important reason)

☐1 Need for accommodation
☐2 Economic consequences
☐3 Collaboration/interaction with colleagues
☐4 Collaboration/interaction with others
☐5 Increased workload for colleagues
☐6 Work capacity
☐7 Other: ____

c) Do you have any experience with colleagues/employees like Matthew?

☐1 Yes
☐2 No

Sarah

Sarah is a 33-year-old mother who has the required qualifications and is in normal good health. She recently finished her maternity leave after having her second child, with the first one being three years old. Sarah is divorced and shares custody for both children with her previous partner. Both children are attending kindergarten. As most parents of young children, Sarah sometimes struggles to make time for everything, often gets the common cold and occasionally has to stay at home with sick children.

a) Given the current circumstances, how do you think Sarah fits into your work group?

Very poorly	Quite poorly	Neither poorly nor well	Quite well	Very well
☐1	☐2	☐3	☐4	☐5

b) If Sarah does not fit quite/very well into your work group: What is the main reason? (Please select the one most important reason)

☐1 Need for accommodation
☐2 Economic consequences
☐3 Collaboration/interaction with colleagues
☐4 Collaboration/interaction with others
☐5 Increased workload for colleagues
☐6 Work capacity
☐7 Other: ____

c) Do you have any experience with colleagues/employees like Sarah?

☐1 Yes
☐2 No

John

John is a man in his forties who has the qualifications needed. He is out of shape, but otherwise has a normal work capacity. He is 5.6 ft. tall and weighs 220 pounds. He is not fond of working out, but since he is classified as moderately to seriously obese his doctor has tried several times to encourage him to be more physically active and eat healthier. John smokes 10 cigarettes a day and does not have any immediate plans of quitting.

a) Given the current circumstances, how do you think John fits into your work group?

Very poorly	Quite poorly	Neither poorly nor well	Quite well	Very well
☐1	☐2	☐3	☐4	☐5

b) If John does not fit quite/very well into your work group: What is the main reason? (Please select the one most important reason)

☐1 Need for accommodation
☐2 Economic consequences
☐3 Collaboration/interaction with colleagues
☐4 Collaboration/interaction with others
☐5 Increased workload for colleagues
☐6 Work capacity
☐7 Other: ____

c) Do you have any experience with colleagues/employees like John?
☐[1] Yes
☐[2] No

Melissa

Melissa is a 38-year-old woman who has the qualifications needed for the job. She goes to see her general practitioner often, and has been experiencing bodily aches and pains for as long as she can remember. She has had periods of chest pain, tenderness in her joints, dizziness and irregular menstruations. Sometimes she worries that her aches and pains may be signs of cancer or other serious disease. Her medical history is long and complex, and several specialists have examined her thoroughly without finding any organic cause of her problems. Melissa's symptoms vary, and in periods where other parts of her life are going well she may feel completely healthy.

a) Given the current circumstances, how do you think Melissa fits into your work group?

Very poorly	Quite poorly	Neither poorly nor well	Quite well	Very well
☐[1]	☐[2]	☐[3]	☐[4]	☐[5]

b) If Melissa does not fit quite/very well into your work group: What is the main reason? (Please select the one most important reason)
☐[1] Need for accommodation
☐[2] Economic consequences
☐[3] Collaboration/interaction with colleagues
☐[4] Collaboration/interaction with others
☐[5] Increased workload for colleagues
☐[6] Work capacity
☐[7] Other: ____

c) Do you have any experience with colleagues/employees like Melissa?
☐[1] Yes
☐[2] No
